# Practical Aspects of Interface Application in CPAP Treatment

**DOI:** 10.1155/2019/7215258

**Published:** 2019-11-03

**Authors:** Adel Bachour, Heidi Avellan-Hietanen, Tuula Palotie, Paula Virkkula

**Affiliations:** ^1^Sleep Unit, Heart and Lung Centre, Helsinki University Hospital, Clinicum, Faculty of Medicine, University of Helsinki, Helsinki, Finland; ^2^Oral and Maxillofacial Diseases, Helsinki University Hospital and Department of Oral and Maxillofacial Diseases, Clinicum, Faculty of Medicine, University of Helsinki, Helsinki, Finland; ^3^Department of Otorhinolaryngology, Head and Neck Surgery, University of Helsinki and Helsinki University Hospital, Helsinki, Finland

## Abstract

While continuous positive airway pressure (CPAP) is an effective first-line therapy for sleep apnea, CPAP fails in one third of patients mainly due to poor adherence to the CPAP device and masks. The role of the medical team is to guide the patient in choosing the best mask, thus insuring good CPAP therapy adherence. Once a suitable mask is found, the brand of the mask does not affect patient satisfaction or CPAP adherence. For the majority of patients, nasal masks are by far more suitable than oronasal masks. Orosanal masks are indicated in case of nasal stuffiness or when an air leak manifests through the mouth. Re-evaluation of the efficacy of CPAP therapy is recommended when switching to oronasal masks.

## 1. Introduction

Continuous positive airway pressure (CPAP) therapy is considered the standard treatment for obstructive sleep apnea (OSA) [1]. CPAP consists of a pump that blows air into the patient's nose, mouth, or both during sleep to hold the airways open and to avoid obstructions. A connecting hose and “mask,” which rests on the patient's face, connects the pump to the patient [2].

Despite numerous improvements in the technology of CPAP masks and devices, the major challenge for physicians is to increase patient acceptance of and adherence to CPAP treatment [3]. About 35% of patients stop CPAP therapy within 1 year [4]; after which the yearly discontinuation rate drops to 3.7% (personal data).

One of the reasons for discontinuing CPAP therapy are problems related to masks. Here we review the conditions for finding an optimal mask, describe mask-related problems and their management, and discuss the effects of these problems on CPAP therapy adherence.

## 2. Types of CPAP Masks

**Figure 1:**
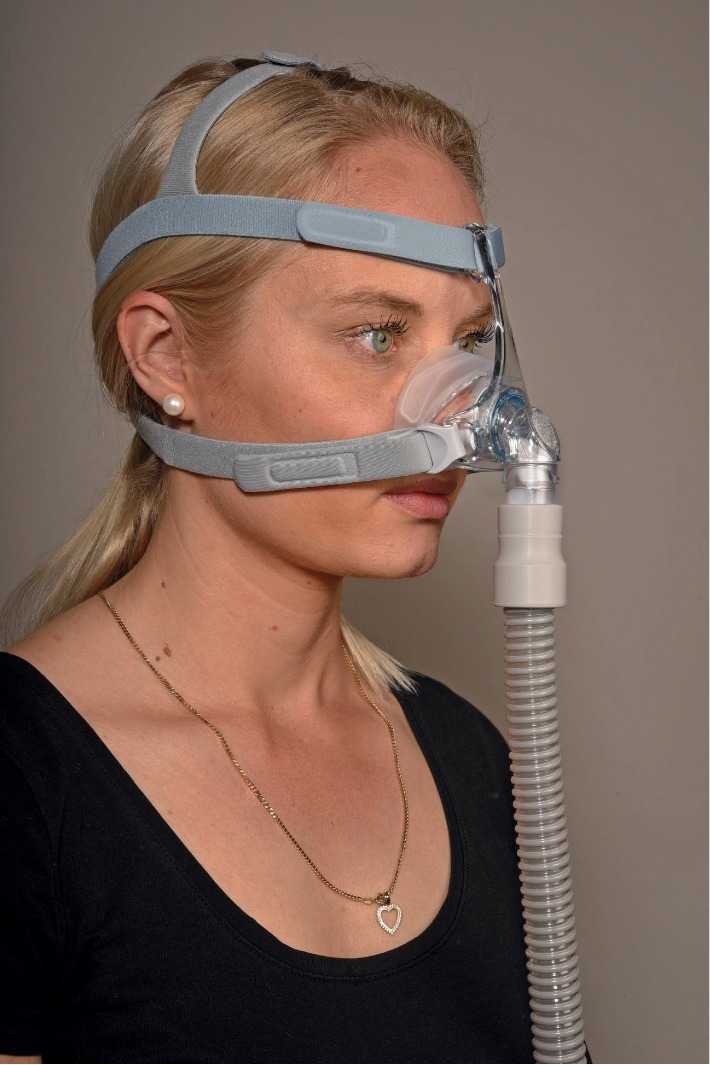
Nasal mask with straps.

**Figure 2:**
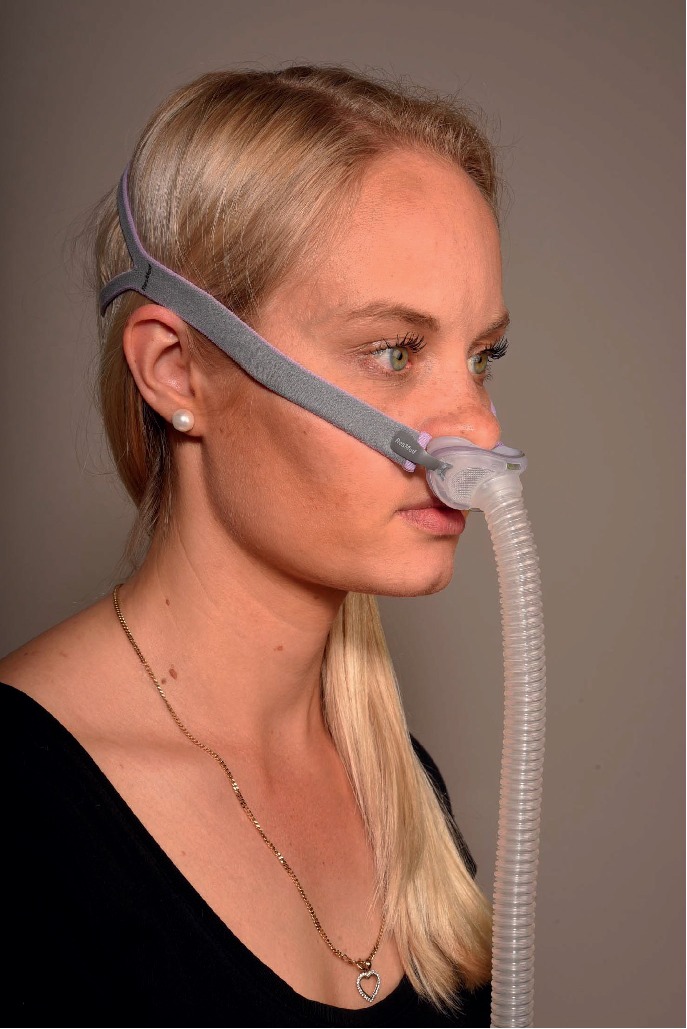
Nasal pillows mask.

**Figure 3:**
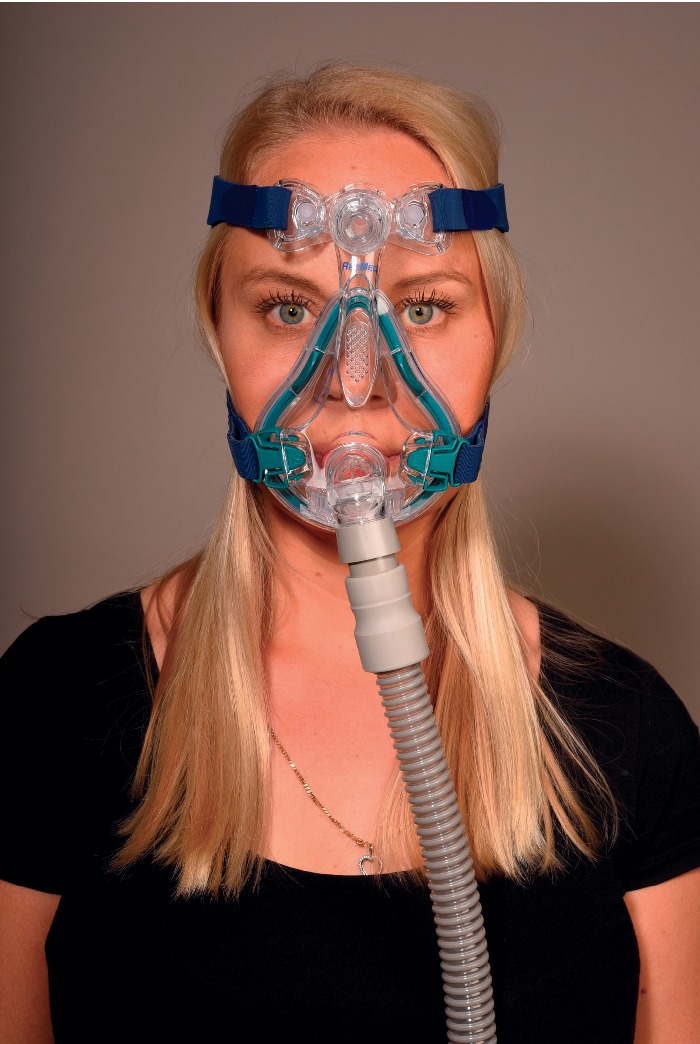
Oronasal mask.

Commercially available masks are masks that cover the nose (nasal masks, Figure 1), masks that fit into the nostrils (nasal pillows masks, Figure 2) or the mouth (oral mask), fit into both the nose and mouth (oronasal or full-face masks, Figure 3), or even cover the entire face (helmet) [5, 6].

About 80% of our patients, use nasal masks followed by oronasal (11%) and nasal pillows masks (9%) [5]. Helmet or oral mask use is rare.

## 3. Characteristics of CPAP Masks

### 3.1. Nasal Mask

The nasal mask is fixed over a solid base covering the areas from the bridge of the nose down to the upper lip. This upper part of the skull is stable and is not affected by respiration, swallowing, phonation, or chewing. The nasal mask has a sufficient support and a good contact with the skin from different directions, thus making it considerably stable. This mask requires good nasal breathing and facial symmetry. It is not recommended in case of facial anatomical deformations, facial paralysis, or diffused skin anomalies of the face.

### 3.2. Nasal Pillows Mask

A nasal pillows mask (Figure 2) has two soft nasal tubes that insert into the nostrils and is secured by straps that go around the head. This mask has the least contact with the skin and is therefore the first choice in case of skin allergy or other anomalies. It is the least bulky among CPAP masks and is easy to carry and is practical for travel. It may also allow the patient to wear glasses and read before falling asleep, as it offers a better field of vision than many of the other mask types. A nasal pillows mask is in general unstable and prone to induce air leaks. However, this mask is suitable for users with beards or moustaches. The lightweight and minimal design is also ideal for patients suffering from claustrophobia.

A nasal pillows mask may not suitable when high CPAP pressure is needed. In addition, the airflow through the nostril is very direct and may cause discomfort at higher pressure settings.

### 3.3. Oronasal Mask

An oronasal mask (Figure 3) is also called a full-face mask. This mask covers the nose and mouth and all or part of the face. Side straps keep the mask in place. Some oronasal masks cover the mouth but also have nasal prongs that fit into the nostrils, similar to a nasal pillows mask. Oronasal masks are indicated in patients with nasal obstruction, as it also allows ventilation through the mouth. Oronasal masks are prone to air leaks as they have a larger surface area and because the lower part of the mask fits over the jaw (a movable part of the face). The second major indication of oronasal masks is in patients with considerable mouth breathing, or in those with an air leak through the mouth in patients using a nasal mask. Oronasal masks are bulky and considerably costlier than nasal or nasal pillows masks. To reduce or prevent lung aspiration and to avoid suffocation in case of defects in the CPAP blowing turbine, oronasal masks are contraindicated in patients with a high regurgitation risk and in patients who are unable to take a CPAP mask off without assistance.

## 4. Measuring Optimal Fit

Finding the right CPAP mask is crucial for success of CPAP therapy. Possible use of an occlusal splint for dental bruxism or orthodontic appliances should also be kept in mind when choosing the CPAP mask. The mask of choice in these patients seems to be a nasal or a nasal pillow mask. These masks might minimize the side effects of simultaneous use of these appliances. In addition, an occlusal splint might even worsen the apnea and hypopnea index (AHI) by additional upper airway narrowing during sleep, although this effect has not been shown to be statistically significant [7].

**Figure 4:**
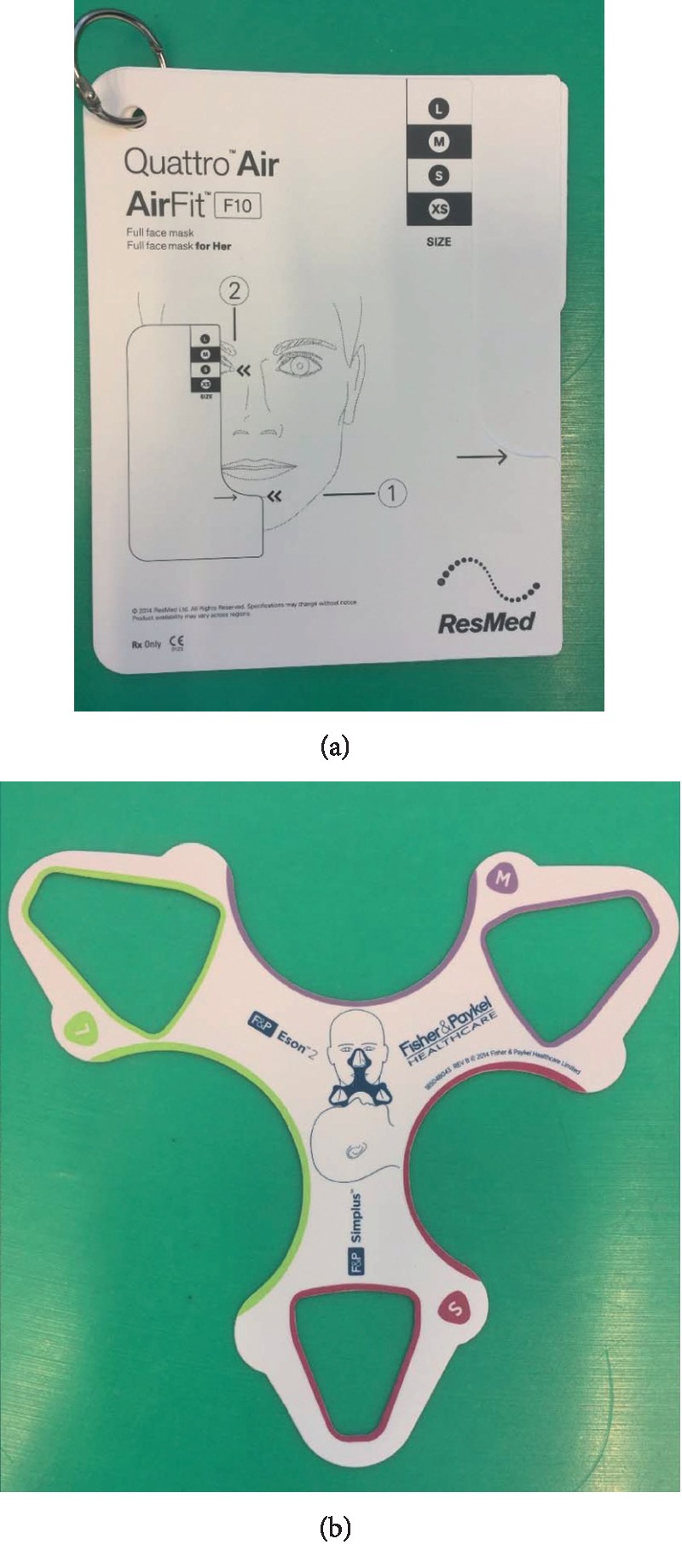
Tools provided by mask manufacturers to help the medical team choose a suitable mask size.

With the aid of measuring tools (Figure 4), the sleep nurse can choose the most suitable mask. The nurse makes subsequent adjustments on the basis of patient feedback. Adjustments are preferable when the patient is in supine position.

While the CPAP device is on, the nurse checks for air leaks and adjusts the mask accordingly again.

The patient is instructed to tighten or loosen the straps as necessary.

Most masks have a cushion to insure a good seal without pressuring the skin due to suction forces created by the air pressure.

## 5. First-Line Mask

A first-line mask policy means that all patients start CPAP therapy with a single mask model (called the first-line mask) and switches masks later when needed. In theory, this policy has the advantage of facilitating logistical needs, simplifying patient preparation, and reducing costs. However, not every patient's specific needs are satisfied in the earliest stage of CPAP therapy under a first-line policy. The definition of first-line mask policy could also apply to a practice that uses a limited choice of masks, such as one of each type.

A recent study by our team has shown an initial CPAP acceptance rate of 81% [2]. This means that 81% of patients continued the same mask beyond 1 year from CPAP initiation and only 19% of patients requested a mask switch.

## 6. Comfort

There is no superiority in terms of comfort between nasal, pillows, and oronasal masks provided that the mask is suitable for the patient. A comfortable mask is one that causes the least disturbances.

## 7. Satisfaction

In a questionnaire study on 730 CPAP users, patients reported a mean satisfaction rate of 68% (0 = absolutely unsatisfied to 100 = very satisfied). This satisfaction rate was not influenced by the type or brand of the mask, previous mask experience, or the age or gender of the patient [5].

## 8. Side Effects of Masks

### 8.1. Dental Changes

Some cephalometric studies have shown non-significant dental and craniofacial changes in long-term CPAP users. None of the studied patients self-reported any permanent change of occlusion or facial profile [8, 9]. These side effects may manifest when the lower mask straps are too tight. Patients sometimes present with sore teeth and lips. The best solution to this problem is to avoid excessive tightening of the lower straps and to adjust them such that they are just tight enough to insure a good seal. Switching to a nasal pillows mask may also help. It is important to note that the existence of progressive dental malocclusion (i.e. mandibular protrusion and open bite) in nasal mask users may also be associated with a very rare systemic disease (such as acromegaly) that should be excluded [10]. Compared to CPAP therapy, oral-appliance therapy for sleep apnea has a slightly higher risk for craniofacial or dental changes. Therefore, patients treated with an oral appliance but also with CPAP therapy need a thorough follow up by a dentist or dental specialist experienced in the field of dental sleep medicine [11].

### 8.2. Local Skin Problems

1. *Morning Print*. A morning print is a consequence of the continuous application of pressure caused by the mask or straps. The applied pressure locally reduces edema and the print takes the shape of the mask or straps. The print appears when the mask is removed in the morning [5]. Any fluid that has accumulated in the lower extremities while standing upright during the day could shift rostrally into the neck and face on assuming the recumbent position during sleep. Such fluid displacement could cause distension of the great veins, edema, or both [12, 13]. The amount of displaced fluid can explain the degree of edema, but the amount of applied pressure explains the variability of its prevalence between different brands of masks [5]. Morning print is not related to the type of mask or to patient age or gender [5].
2. *Skin Pressuring.* About half of patients experience CPAP mask pressure on the skin, especially oronasal mask users [5].
3. Minor skin lesions (such as redness or erosion) were reported in one fifth of patients, especially on the lateral sides of the nose and on the nasal bridge [5].

### 8.3. Sleep Position

It is a reasonable assumption that use of CPAP masks would prevent sleeping in prone position. Although there are no studies indicating the prevalence of prone sleep position, use of CPAP mask completely changes the sleep position in one fourth of patients.

## 9. Maintenance of CPAP Masks

The patient should follow the advice of the medical team, the medical providers, and the manufacturer's instructions for cleaning the masks. Keeping the mask clean and dry insures long-time use and optimal fit.

## 10. Nasal versus Oronasal Mask

**Figure 5:**
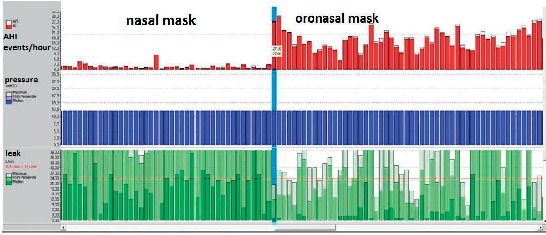
A screenshot from a ResScan program (ResMed, San Diego, California, USA) showing 2 months of CPAP therapy with a fixed pressure at 12 cm H_2_O. To reduce leaks, the nasal mask was switched to an oronasal mask, which led to a decrease in leak values (dark green) but to an increase in AHI (red and white). Oronasal masks require effectively higher CPAP pressures than nasal masks.

The CPAP pressure through the nasal mask pushes the soft palate and tongue forward and keeps the upper airways open. Although the oronasal mask violates this principle, previous studies have shown that oronasal CPAP can treat obstructive sleep apnea [2, 14, 15]. Oronasal masks have a negative impact on CPAP adherence, therfore a nasal mask should be preferred as the first option [16]. A recent meta-analysis has shown that oronasal masks are associated with higher CPAP levels, higher residual AHI scores (Figure 5), and poorer adherence than nasal masks [15].

## 11. Air Leak

**Figure 6:**
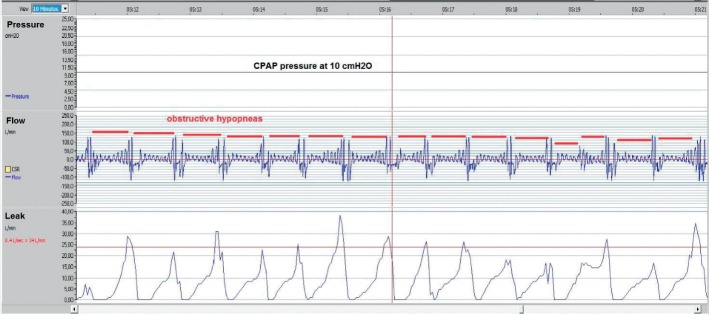
A 10-minute screenshot from the ResScan program for CPAP follow up. In the lower panel, we see an intermittent air leak that caused instantaneous pressure drop in the upper airways and led to obstructive hypopneas.

Air leaks should be checked at every medical visit for CPAP therapy. Leak values and graphics are available by connecting the CPAP device to a computer running specific software (Figure 6). The CPAP device shows values of instantaneous leaks and those of the whole night.

An air leak is one of the major problems related to CPAP therapy. There are two types of leaks, namely intentional and unintentional.

An intentional leak is intended to rinse the air in front the nose or the mouth and therefore to avoid rebreathing. The minimal pressure needed is 4 cm H_2_O. All masks have a vent to ensure intentional leaks. This vent should always be functional. As the both intentional and unintentional leaks increase with increase in CPAP pressure [17]; recent CPAP devices calculate the total leaks and extract intentional leaks. This allows reporting of only unintentional leaks, which should be 0 liter/minute in optimal conditions.

An unintentional leak (here simply called “leak”) may manifest but remain unnoticed by the patient (no disturbing leak) or could causes some disturbances, such as increased noise (due to an increase in turbine rotation to compensate for the leak or from the air turbulance in the CPAP tube) disturbing the bed partner, feeling cool air against the body or the face, mouth drying, or nasal stuffiness or watering. Air could also escape to the patient's stomach and cause abdominal symptoms. This latter “interior leak” is not the subject of this paper.

## 12. Reasons for Unintentional Leaks

Leaks may be due to an unfit mask, unsealed tubing, or an unsealed humidification circuit. Leaks can also emerge through the lacrimal canals or the mouth.

### 12.1. Unfit Mask

An unfit mask should be adjusted or switched to another one. Occasionally, the mask is placed very tightly, which places the sealing cushion in a non-functional position.

### 12.2. Unsealed Tubing

It is important to regularly check the tubing for leaks. If leaks persist when the tubing outlet is completely blocked while the CPAP device is blowing out, then the tube or the humidificator are not well sealed. Replacing the tube may resolve the leak.

### 12.3. Unsealed Humidification Circuit

Tight sealing can be verified by taking the tube off and then obstructing the air coming out from the humidificator. If the leak persists despite complete obstruction, the cover of the cover humidificator should be checked or the whole humidification circuit should be replaced if necessary.

### 12.4. Leak through the Lacrimal Canals

A leak through the lacrimal canals usually manifests by complaints of eye redness and watering, especially in patients with a history of nasolacrimal surgery. The amount of leak may be very low and remain unnoticed in the leak report. A bubbling test is recommended to confirm the diagnosis [18]. This test consists of applying a few drops of physiological saline to the patient's eyes while they are in supine position. The patient should be awake and the CPAP device should be on. An air leak is confirmed when air emerges as bubbles from the lower lacrimal punctum. The test should be performed in both eyes. Treatment consists of inserting dissolvable or permanent (silicone) lacrimal plugs into the corresponding lower lacrimal puncta.

### 12.5. Air Leak through the Mouth

**Figure 7:**
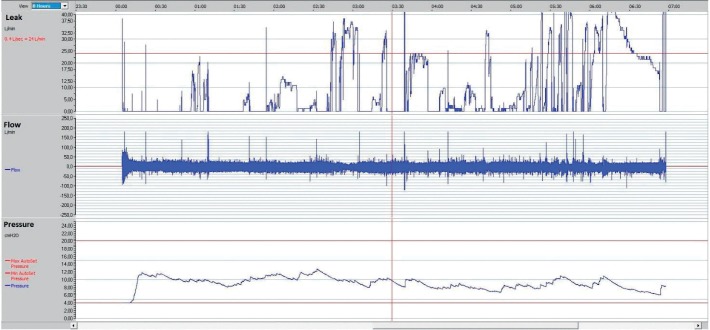
Notice the sawtooth shape of the leak curve in the upper right side. This indicates an abnormal and intermittent leak with a normalization of the leak between leak episodes. The sawtooth shape is usually caused by a mouth leak, as mouth leaks are never continuous. Such leaks dry the mouth and force the patient to close their mouth to restore mouth humidification.

If the patient has a nasal pillows or nasal mask, air may leak when the patient opens their mouth. This leak is unidirectional, namely the air comes in through the nose and out through the mouth. If persistent, this leak usually causes mouth dryness and nasal stuffiness. The latter may increase mouth leak as nasal breathing is disturbed, causing a vicious circle. During sleep, the jaw may drop slowly down leading to opening of the mouth. The leak then increases progressively until the patient closes their mouth to stop the leaks. These short episodes of high leaks give the leak curve a sawtooth shape (Figure 7). To break this vicious circle, it is important to restore nasal breathing (using humidification) or to switch to an oronasal mask. Chinstraps may also be useful (see below). Patients with a history of uvulupalatopharyngoplasty usually need oronasal masks to avoid mouth leak. As the soft palate is removed in these patients, the expired air could easily reach the mouth cavity and escape through the mouth. Leaks through the mouth may also occur with an oronasal mask. These leaks manifest when the lower part of the mask is loose. Tightening the lower part may push the jaw backward, leading to high CPAP pressure needs and therefore higher leak values.

**Figure 8:**
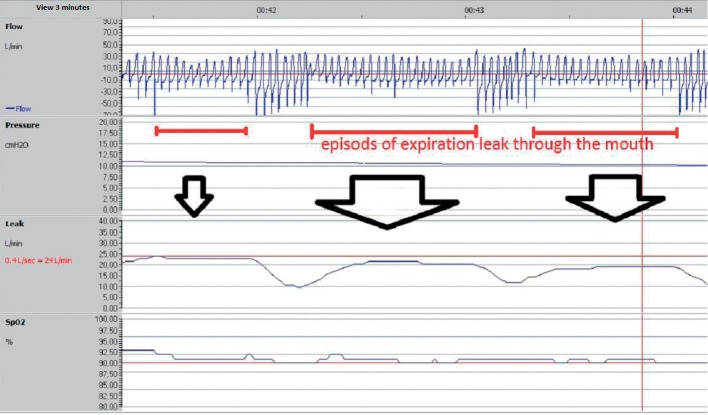
A 3-minute recording of CPAP therapy showing three episodes of expiration leak through the mouth. The respiration flow.

When the mouth leak occurs only during expiration, the respiration airflow curve shows an amputation of the expiratory part of the curve associated with a slight increase of the leak debit (Figure 8). This leak during expiration occurs especially in the supine position, as the soft palate may move backward thus blocking the expiratory nasal pathway and directing the expired air through the mouth.

## 13. Outcomes of Switching Masks

About 19% of new CPAP patients switch their masks during the first year of therapy. This mainly occur within 2 weeks from CPAP initiation. After the first year, mask switches are rare and the mean interval time is 66 months [2]. We previously reported that women switched their CPAP masks 1.2 times more often than men, probably because masks were initially designed for men [2]. Recently, several masks designed specifically for women have become available.

The major reason for mask switching was poor fit or discomfort; this occurred in more than one third of cases of switching. Switching a poorly fitting mask resolved the problem in about two thirds. The second major reason for switching was that the masks were outdated. In this cas, the new mask was suitable for 80% of cases [2].

## 14. Management of Air Leak

**Figure 9:**
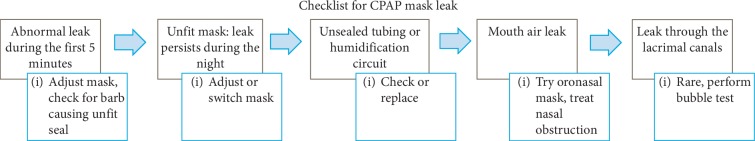
Checklist for managing mask leak.

The best way to avoid air leaks is to find the most suitable mask at the CPAP initiation period. However, when leaks emerge, the patient is asked to check the mask fit and adjust the straps. The sleep nurse can check leak values and their effects on CPAP therapy. The CPAP device can compensate for low leaks without therapeutically significant consequences. We previously reported that 65% of our patients experienced a disturbing leak [5]; but only 0.1% of patients requested a mask switch [2]. Switching to an oronasal mask or nasal pillows mask can help reduce leaks caused by mouth air leak. Sometimes switching to a different mask brand can help reduce leaks. It is important to ask the patient to wear the mask during the outpatient visit to ensure correct fitting. Treating nasal stuffiness is also important in preventing leaks.  Figure 9 showes practical step to manage leak.

**Figure 10:**
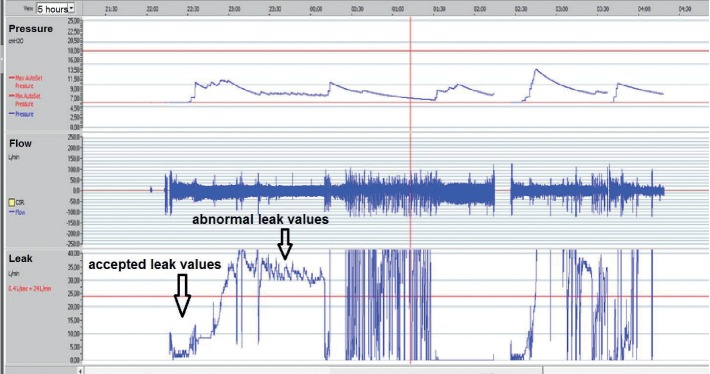
Screenshot of a 5-hour CPAP therapy showing that leak values were acceptable (below 5 liters/minutes) at the beginning of the night and later leak values increased above the critical threshold (24 liters/minutes).

In practice, high leaks at the beginning of the night can be easily reduced by adjusting or switching the mask. Leaks that emerge later are more difficult to resolve, as they are not related to defects in mask adjustment (Figure 10).

### 14.1. Chinstrap

**Figure 11:**
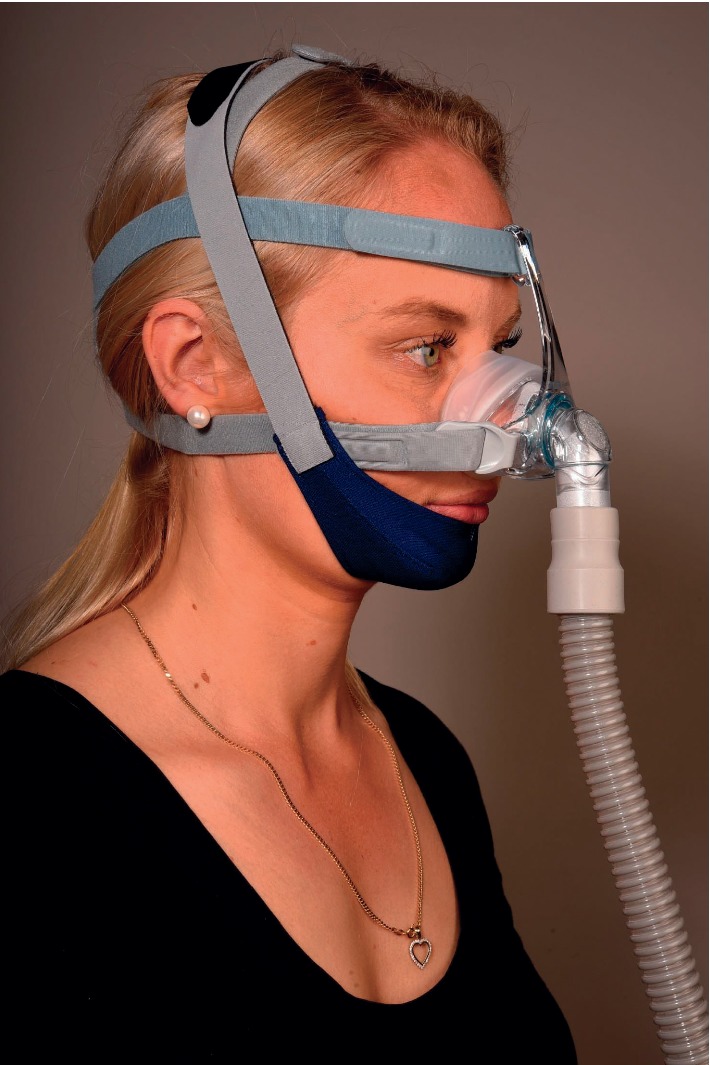
The chinstrap prevents the mouth from opening during CPAP therapy.

The chinstrap prevents the patient's mouth from opening and is placed around the chin and attached to the headgear of the mask (Figure 11).

By closing the mouth during CPAP, the chinstrap reduces mouth leaks and moderately reduces the arousal index in most patients [19]. The chinstrap may also increase snoring by pushing the jaw backward, thus reducing the upper airway space [19]. In rare cases, the chinstrap can worsen the respiratory disturbance index [19]. It is important to adjust the upper part of the straps as forward as possible to reduce the backward pushing force of the chinstrap.

## 15. Benefits to Adherence

We previously reported that users of nasal interfaces used their CPAP daily significantly one hour more than users of nasal pillow or oronasal interfaces [5]. Borel et al. have reported that the type of mask influences CPAP adherence. These results were recently confirmed by a meta-analysis study that showed the superiority of nasal masks over oronasal masks in terms of CPAP adherence [15]. Failure to provide humidification to patients with initial nasal symptoms may lead to low CPAP adherence or even CPAP cessation [20].

## 16. Conclusion

Choosing suitable CPAP masks is an important part of CPAP therapy acceptance and adherence. All efforts should be directed towards reducing discomfort and leaks. Several rules should be followed to control leaks, insure good acceptance, and obtain better CPAP adherence.

## Abbreviations

AHI: Apnea and hypopnea index
CPAP: Continuous positive airway pressure
OSA: Obstructive sleep apnea.
